# Tumor-educated B cells promote renal cancer metastasis via inducing the IL-1β/HIF-2α/Notch1 signals

**DOI:** 10.1038/s41419-020-2355-x

**Published:** 2020-03-02

**Authors:** Saiyang Li, Chi Huang, Guanghui Hu, Junjie Ma, Yonghui Chen, Jin Zhang, Yiran Huang, Junhua Zheng, Wei Xue, Yunfei Xu, Wei Zhai

**Affiliations:** 1 Department of Urology, Shanghai Tenth People’s Hospital, Nanjing Medical University, 211166 Nanjing, China; 2 Department of Urology, the Affiliated Changzhou Maternity and Child Health Care Hospital of Nanjing Medical University, 16 DingxiangRoad, Zhonglou District, 213000 Changzhou, Jiangsu China; 3 Department of Urology, Shanghai Tenth People’s Hospital, School of Medicine in Tongji University, 200072 Shanghai, China; 4 Department of Urology, Shanghai Pudong Hospital, Fudan University Pudong Medical Center, 201399 Shanghai, China; 5 Department of Urology, Renji Hospital, School of Medicine in Shanghai Jiao Tong University, 160 Pujian Road, Pudong District, 200127 Shanghai, China; 6 Department of Urology, Shanghai First People’s Hospital, School of Medicine in Shanghai Jiao Tong University, 200080 Shanghai, China

**Keywords:** Renal cell carcinoma, Cell invasion

## Abstract

While B cells in the tumor microenvironment (TME) might play important roles in cancer progression, their impacts on the renal cell carcinoma (RCC) metastasis remained unclear, which drew our attention to further explore. We found that RCC tissues could recruit more B cells than the surrounding normal renal tissues from human clinical RCC samples. Wound healing assay, transwell assay and 3D invasion assays demonstrated that recruited B cells, also known as tumor-educated B cells (TEB), could significantly increase the RCC cell migration and invasion. In addition, in vivo data from xenograft RCC mouse model also confirmed that TEB could enhance RCC cell invasive and metastatic capability. Mechanism dissection revealed that TEB activated IL-1β/HIF-2α signals in RCC cells that could induce the downstream Notch1 signaling pathway. The above results demonstrated the key roles of TEB within renal cancer associated tumor microenvironment were metastasis-promotor and might help us to develop the potential therapies via targeting these newly identified IL-1β/HIF-2α/Notch1 signals in RCC progression.

## Introduction and objectives

Accumulating evidence revealed that tumor microenvironment (TME) was critical in renal cell carcinoma (RCC) progression^1–3^. Intriguingly, the mean frequencies of B lymphocytes (B cells) in immune cells from the RCC TME were 4%^4^. Yet, as a crucial component of TME, the role of B cells during RCC metastasis remained sealed. It was widely recognized that B cells could differentiate into plasma cells and produce antibodies after antigen stimulation^5–7^. Nevertheless, it was reported that tumor-educated B (TEB) cells contributed to tumor heterogeneity and therapy resistance in melanoma^8^, and participated in bladder tumor progression^9^. Another study found that tumor-educated B cells selectively promote breast cancer lymph node metastasis by HSPA4-targeting IgG^10^. These findings supported the hypothesis that educated B cells by tumor and cytokines released from tumor established a stable microenvironment, which made it easier for tumor cells metastasis and colonization. These studies promoted us to explore TEB cells’ functional significance in metastasis renal cell carcinoma (mRCC).

Cytokines and growth factors were critical components of RCC TME^11^. Particularly, inflammatory cytokines produced by B cells were strongly linked with development and progression of various carcinomas^12^. As previously reported, interleukin 8 (IL-8) could increase bladder cancer metastasis^9^ and interleukin 6 (IL-6) was associated with poor prognosis in patients with esophageal cancer^13^. Notably, interleukin-1β (IL-1β) could promote hypoxia-induced apoptosis in glioblastoma cells^14^. There were also some studies suggesting that IL-1β got implicated in metastasis of RCC^15^, but whether TEB promoted RCC metastasis via the IL-1β and how IL-1β acted as a metastasis-promotor remained unknown.

RCC was associated with loss of the von Hippel-Lindau (VHL) suppressor protein and some other chromatin-modulating factors^16,17^. VHL was an ubiquitin E3 ligase that targeted different hypoxia inducible factor (HIF) isoforms (HIF-1α and HIF-2α) for proteasome degradation in an oxygen-level-dependent manner^18,19^. In addition, our team used to confirm that HIF-2α acted as an oncogene in RCC and promoted renal cancer cell proliferation under hypoxia^20^. However, whether HIF-2α was implicated in the communication between RCC TME and TEB still remained unclear.

The Notch pathway had been linked to a variety of solid and hematopoietic tumors, and most studies had confirmed a tumor promotional role for the pathway^21–23^. Up to now, four Notch genes (Notch1-4) had been determined in mammals, which encoded receptors for five Notch ligands (Delta1, 3, 4; Jagged1, 2)^24^. The Notch receptors and ligands comprised a conserved, widely expressed family of single-span transmembrane polypeptides that mediate various cellular processes including proliferation, migration, invasion and apoptosis via direct cell-cell contact^25^. Interestingly, Notch may act as either an oncogene or a tumor suppressor gene in the progress of cancer^26^.

In this study, we reported that B cells could be recruited more easily into the RCC tissues compared to the surrounding normal renal tissues in renal cancer associated TME. Increased infiltrating B cells to RCC could promote RCC cell metastasis via modulation of IL-1β/HIF-2α/Notch1 signals.

## Materials and methods

### Clinical samples and cell culture

Tumor samples and paired normal tissues from 20 RCC patients were obtained from the Department of Urology, Shanghai Tenth People’s Hospital, Tongji University (Shanghai, China). The current study was approved by the ethics committee of Shanghai Tenth People’s Hospital. The human RCC cell lines, 786-O and OSRC-2, were originally purchased from Cell Bank of the Chinese Academy of Sciences (Shanghai, China). Both cells were cultured in RMPI 1640 (Gibco, Grand Island, New York, USA) plus 10% fetal bovine serum (FBS, Hyclone, Logan, Utah, USA) with 1% penicillin/streptomycin (P/S, Gibco, Grand Island, New York, USA).

### B cells isolation and culture

B cells were purified from the peripheral blood of healthy donors using anti-CD19 MACS beads (MiltenyBiotec, Auburn, CA) according to the manufacture’s instructions. The supernatant of the co-cultured cells and B cells were collected for further experiments.

### Wound healing assay

To analyze the migration, indicated cells were co-cultured with B cells for 72 h, and then co-plated in six-well plates. Streaks across the plate were created in the monolayer with a pipette tip. Progression of migration was observed and photographed at 0 and 24 h after wounding. The data shown were representative micrographs of wound healing assay of the indicated cells.

### Transwell assay

RCC cells were co-cultured with B cells for 72 h and then plated into the upper layer. RCC cells invaded to the lower surface of the polycarbonate membrane coated with Matrigel through the 8.0-μm pore. Progression of invasion was observed and photographed at 0 and 24 h after plating. The data shown were representative micrographs of transwell assay of the indicated cells.

### 3D invasion assay

Matrigel was thawed on ice and added to each well of six-well plates (at 50 μl/cm^2^), was spread evenly and the plates placed in the cell culture incubator to allow the Matrigel to solidify (takes 15–20 min). RCC cells were placed into each well after co-culture with B cells for 72 h. OSRC-2 and 786-O cells spent about 8 days in forming acini-like structures. The data shown were representative micrographs of 3D invasion assay of the indicated cells.

### Xenograft studies

OSRC-2 cells with firefly luciferase expression were injected intravenously into the tail vein of 6-week-old male nude mice from ShanghaiSipper-BK laboratory animal Company (Shanghai, China). After OSRC-2 cells were injected, B cells were injected 2 h after injecting OSRC-2 cells (*n* = 8 mice/group). Cells were also transduced with luciferase in vivo imaging system that was performed once a week. Studies on animals were conducted with approval from the ethics committee of Shanghai Tenth People’s Hospital.

### Cell transfection

To generate HIF-2α knocked down stable clones, OSRC-2 and 786-O cells were transfected with lentiviral vectors. The cells were transfected using the Lipofectamine 2000 (Invitrogen) reverse transfection protocol, according to the manufacturer’s instructions. The cells were harvested at 48 h after transfection. The stably knocking down cell lines was identified using qRT-PCR.

### Quantitative real-time polymerase chain reaction

Total RNA was extracted from cells using Trizol reagent (Invitrogen). cDNAs were synthesized with PrimeScript RT reagent Kit (Takara, Kusatsu, Japan). Quantitative real-time polymerase chain reaction (qRT-PCR) was performed with KAPA SYBR FAST qPCR Kit (Kapa Biosystems, Woburn, Massachusetts, USA) using a 7900HT Fast Real-Time PCR System (Applied Biosystems, Carlsbad, California, USA). The primers were listed in Supplementary Table S1. The expression levels of mRNA were normalized to endogenous control GAPDH. The 2^−∆∆Ct^ method was used to analyze the expression levels normalized to the endogenous control.

### Western blot analysis

The cells were lysed using RIPA buffer plus protease inhibitors and phosphatase inhibitors. For western blot analysis, 25 μg of protein extracts were loaded to 10% sodium dodecylsulfate–polyacrylamide gel electrophoresis gels and transferred to nitrocellulose membranes. The membranes were incubated with a primary antibody overnight and were incubated with a secondary antibody in 1 h with room temperature. The expression of β-actin was used as loading control. The information of antibodies was listed as follow: CD19: Sino Biological Cat, 11880-R109; CD20: Sangon Biotech, NO.198781; CD40: ABclonal, A13285; HIF-2α: abcam, ab179825; AKT: abcam, ab179463; P-AKT: abcam, ab131443; P65: Cell Signaling Technology, 8242S; P-P65: abcam, ab183559; DLL4: abcam, ab7280; Notch1: abcam, ab52627; Hey1: abcam, ab111723; MMP-9: abcam, ab76003; β-actin: abcam, ab8226.

### Immunofluorescence staining (IF staining)

Tumor sections were placed on slides and were incubated with primary antibodie as well as anti-firefly in 3% bovine serum albumin in phosphate-buffered saline overnight at 4 °C, followed by respective secondary antibodies. The stained slides were mounted and visualized by a fluorescent microscope.

### Enzyme linked immunosorbent assay

Conditioned media (CM) was collected from RCC cells or from co-cultures of RCC cells and B cells for 72 h. CM was used for the detection of cytokines by human cytokines Enzyme linked immunosorbent assay (ELISA) kits according to the manufacturer’s instructions.

### Immunohistochemical staining

Immunohistochemical (IHC) was performed with antibodies specific for CD19, CD20, and CD40 on the samples from the human RCC tissues and paired non-cancerous tissues. The degree of positivity was initially classified according to scoring both the proportion of positive staining cells and the staining intensities.

### Luciferase reporter gene assay

To confirm whether HIF-2α could increase DLL4 promoter activity, 786-O cells transfected with oe-HIF-2α or negative control cultured in 48-well plates were co-transfected with 1.5 μg of firefly luciferase reporter and 0.35 ng Renilla luciferase reporter with Lipofectamine 2000. Luciferase reporter assay used the one step directed cloning kit (Novoprotein, Shanghai, China) according to the manufacturer’s manual.

### Chromatin immunoprecipitation (ChIP)

Cells were crosslinked with 4% formaldehyde for 10 min followed by cell collection and sonication with a predetermined power to yield genomic DNA fragments of 300 bp long. Lysates were precleared sequentially with normal rabbit IgG and protein A agarose. Anti-HIF-2α antibody (2.0 μg) was added to the cell lysates and incubated at 4 °C overnight. For the negative control, IgG was used in the reaction. Specific primer sets designed to amplify a target sequence within human DLL4 promoter were listed in Table S1; PCR products were analyzed by agarose gel electrophoresis.

### Statistical analysis

Results are expressed at least three independent experiments. Using the GraphPad Prism statistical program, data were analyzed using ANOVA or Student’s *t*-test unless otherwise specified. *P* values < 0.05 were considered significant.

## Results and conclusion

### More B cells were recruited in RCC tissues compared with the adjacent normal tissues

**Fig. 1:**
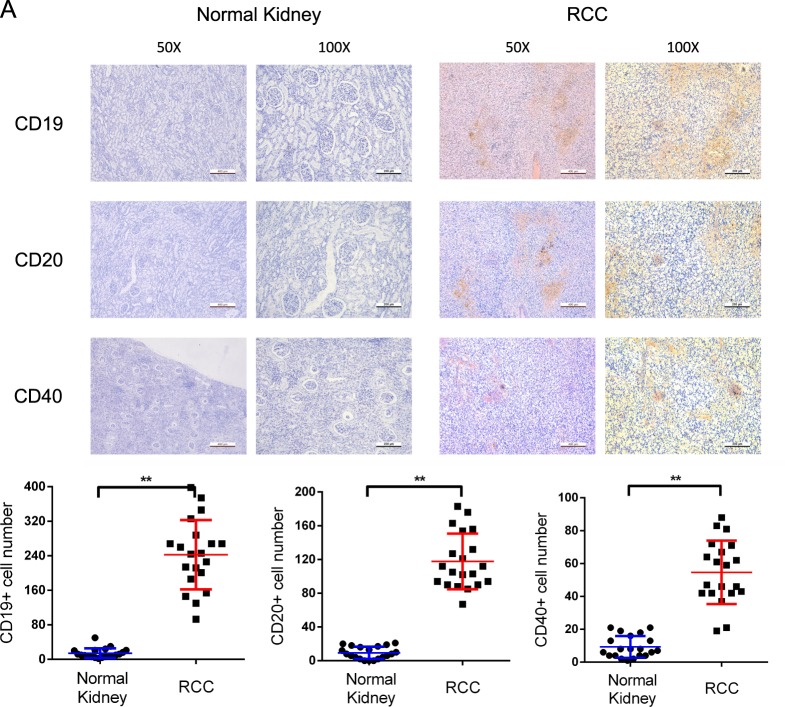
More B cells were recruited in RCC tissues compared with the adjacent normal tissues. **a** Upper panel showed the immunohistochemical staining of CD19, CD20, and CD40 in RCC tumor tissues and adjacent normal renal tissues. In all, ×50 and ×100 magnified images were obtained from an optical microscope. Lower panel was the quantification of CD19, CD20, and CD40 expression. **P < 0.01.

Previous reports indicated that CD20 was a membrane-spanning protein that is present only in B lymphocytes and participate in the differentiation of B cells^27^. Besides, CD19 and CD40 were also important B lymphocyte surface proteins^28,29^. The combination of them could be used as B lymphocyte phenotypic markers to validate the existence of B cells in various human cancer tissues. Therefore, we detected these proteins with IHC staining to confirm the infiltration of B cells in RCC tissue samples. As shown in Fig. 1, more cells were CD19, CD20 or CD40 positive in RCC tissues compared with normal kidney tissues, indicating that B cells were more easily recruited to RCC tissues in renal cancer associated TME.

### B cells facilitated RCC metastasis in vitro and in vivo

**Fig. 2:**
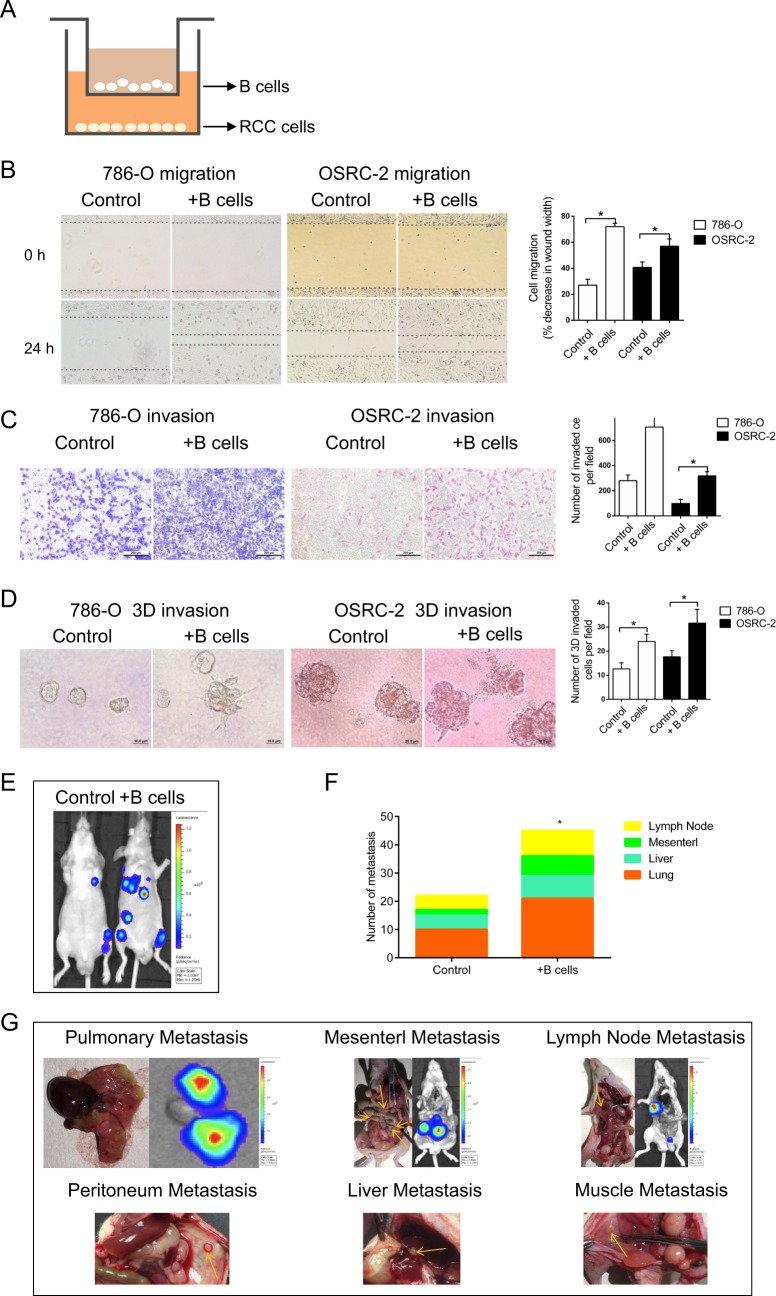

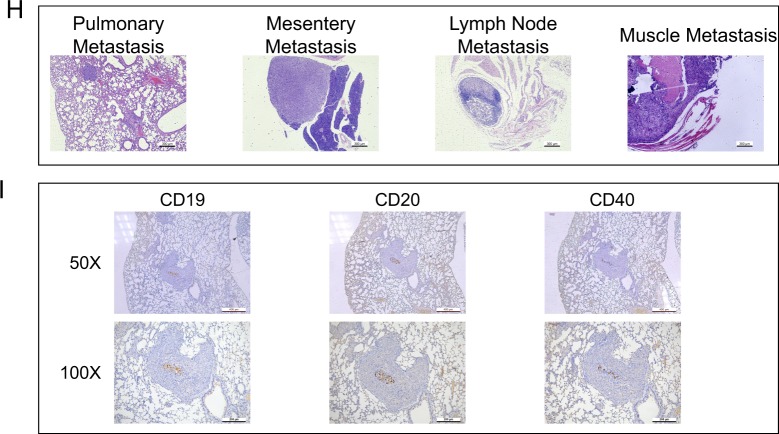
B cells facilitated RCC metastasis in vitro and in vivo. **a** The schematic diagram of the co-culture system. **b** The migration of 786-O and OSRC-2 cells was detected by wound healing assay. **c** The invasion of 786-O and OSRC-2 cells was detected by transwell assay. **d** 3D invasion assay was performed to further evaluate the invasiveness of 786-O and OSRC-2 cells. **e** Representative images of mice viewed by IVIS system in control and co-injected with B cells group 8 weeks after tail vein injection (*n* = 8). **f** Statistics of the number of metastasis nodules in tail vein injected nude mice model established as above. **g** Representative images of mice viewed by IVIS system 8 weeks after tail vein injection. **h** The animals were euthanized 8 weeks later for metastases detection by histological staining with haematoxylin and eosin (H & E). **i** Representative images of the immunohistochemical staining of CD19, CD20, and CD40 in tumor tissues of lung metastasis nodules. *P < 0.05.

B cells were recruited more easily to RCC tissues compared with the adjacent normal renal tissues as shown above. Next, we investigated whether B cells infiltration could alter RCC progression. RCC cell lines were co-cultured with B cells in a co-culture system as shown in Fig. 2a before the migration and invasion assay. Specifically, B cells were seeded into the upper layer of the inserts, whereas RCC cells were seeded into the lower layer. Effector molecules, such as cytokines, could pass through the 0.4-μm pore of the polycarbonate membrane insert. After co-cultured with B cells for 72 h, we first employed wound healing assay to compare the 786-O cells migratory capability with vs without co-cultured B cells. We observed increased migration in 786-O cells, and the similar phenomenon was found when 786-O cells were replaced by OSRC-2 cells (Fig. 2b). In parallel, Transwell assay substantiated that co-culturing RCC cells with B cells significantly enhanced the invasive ability of both RCC cells, respectively (Fig. 2c). Furthermore, 3D invasion assay also elucidated that more cell processes formed in the presence of B cells, indicating the elevated invasive capacity (Fig. 2d).

To confirm the above in vitro results, we established tail vein injected nude mice model with OSRC-2 cells mixed with B cells. The OSRC-2 cells were transfected with firefly luciferase expression for monitoring metastasis using the in vivo real-time imaging system (IVIS) (Fig. 2e). After 8 weeks, IVIS results revealed that in the OSRC-2/B cells co-injected group had more metastatic luminescence signals (Fig. 2f). The mice were then sacrificed for tumor confirmation, and results revealed that the metastatic nodes were exclusively found in lung, abdominal cavity, lymph nodes, peritoneum and liver(Fig. 2g, h). Immunohistochemical staining confirmed high expressed CD19, CD20, and CD40 in representative lung metastasis nodules (Fig. 2i). Taken together, tumor microenvironment recruited B cells could facilitate RCC metastasis not only in vitro but also in vivo.

### B cells promoted RCC cell migration and invasion through the increase of HIF-2α expression and translocation

**Fig. 3:**
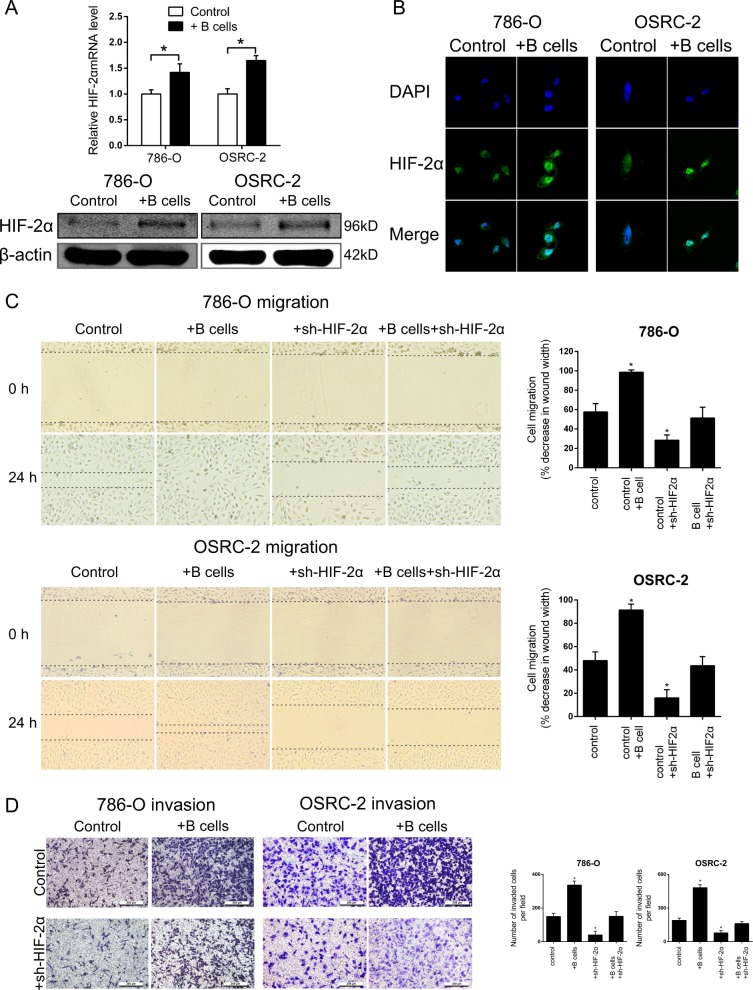
B cells promoted RCC cell migration and invasion through the increase of HIF-2α expression and translocation. **a** After co-cultured with B cells, the mRNA and protein levels of HIF-2α in 786-O and OSRC-2 cells was detected by qRT-PCR (upper panel) and western blot (lower panel). **b** HIF-2α expression was further examined by IF assay. Scale bar = 10 μm. 786-O and OSRC-2 cells were co-cultured with B cells, transfected with sh-HIF-2α or the combination. **c** Cell migration was tested by wound healing assay in both RCC cells. **d** Cell invasion was examined by transwell assay in both RCC cells. *P < 0.05.

Previously, we identified that HIF-2α transcriptionally regulated lncRNA-SARCC expression to affect hypoxic RCC cell proliferation^20^. In this study, we hypothesized that HIF-2α signaling was involved in renal cancer associated TME by which B cells enhance RCC progression due to HIF-2α functioned as an oncogene in RCC. qRT-PCR and western blot demonstrated that HIF-2α increased at mRNA and protein levels in 786-O and OSRC-2 cells after co-culture with B cells (Fig. 3a). Moreover, IF staining showed the elevated fluorescence intensity of HIF-2α in the co-culture groups, suggesting the stimulation of B cells on HIF-2α translocation (Fig. 3b). To further investigate the potential role of HIF-2α in B cell increased migration and invasion. HIF-2α-specific shRNA1 and 2 was introduced to interfere with HIF-2α expression (Supplementary Fig. 1A). As shown in Figs. 3c, d, HIF-2α silencing suppressed the migration and invasion of 786-O and OSRC-2 cells. Notably, B cell increased migration and invasion, which were impaired strikingly by sh-HIF-2α.

Collectively, co-cultured B cells effectively potentiated the migratory and invasive capacity of RCC cells through inducing HIF-2α expression and translocation.

### B cells potentiated HIF-2α expression through the secretion of IL-1β

**Fig. 4:**
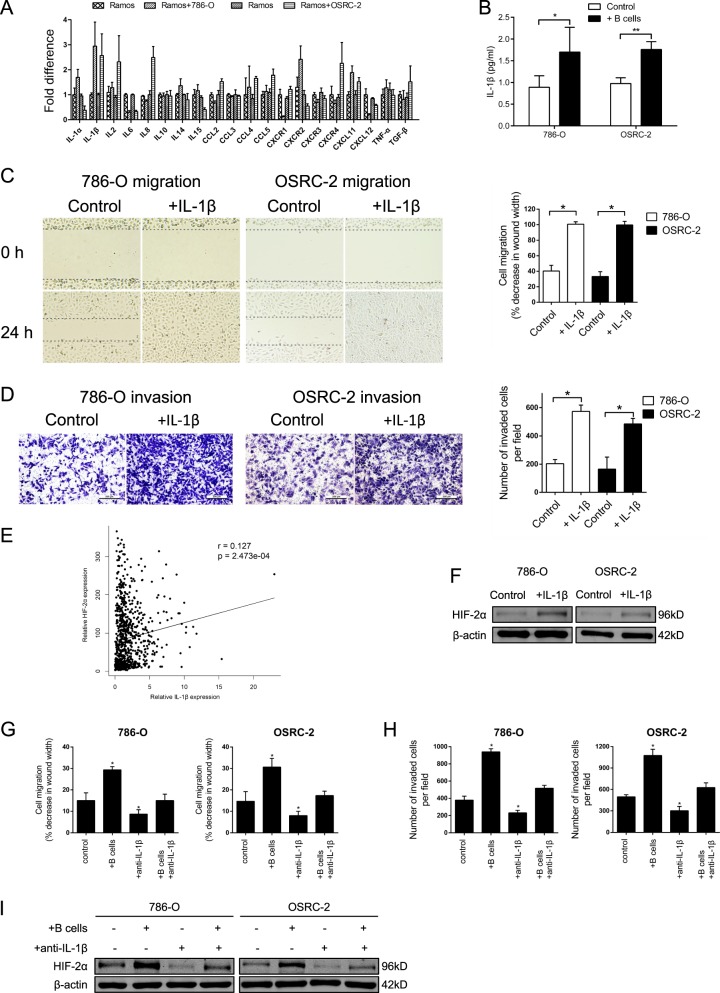
B cells potentiated HIF-2α expression through the secretion of IL-1β. **a** The screening of relevant cytokines that might be altered by the interaction between RCC cells and B cells. **b** The concentrations of IL-1β in the cell culture supernatants of control groups and co-culture groups were detected by ELISA. RCC cell lines were pretreated with recombinant human IL-1β. Wound healing assay (**c**) and transwell assay (**d**) were performed respectively to investigate the effect of ectogenous IL-1β on cell migration and invasion. **e** IL-1β mRNA expression and corresponding HIF-2α mRNA expression in RCC samples from TCGA dataset. **f** HIF-2α expression with the stimuli of IL-1β was detected by WB. 786-O and OSRC-2 cells were co-cultured with B cells, pretreated with IL-1β neutralizing antibody or the combination. Wound healing assay (**g**) and transwell assay (**h**) were performed respectively to assess the capacities of cell migration and invasion. HIF-2α expression was detected by WB (**i**). *P < 0.05, **P < 0.01.

Considering cytokines were the primary mediators by which immune cells exerted regulatory effects, we detected the transcription levels of a list of cytokines related with B cells by qRT-PCR. Results suggested that IL-1β transcription was enhanced in B cells after co-cultured with 786-O and OSRC-2 cells separately (Fig. 4a). In addition, ELISA further validated the increase of IL-1β secretion in the cell culture supernatants of co-culture groups (Fig. 4b). It was potentially conceivable that B cells promoted RCC metastasis through the excessive secretion of IL-1β. Consequently, 786-O and OSRC-2 cells were treated with recombinant human IL-1β instead of co-culturing with B cells. As a result, IL-1β significantly facilitated the migration and invasion of RCC cells (Fig. 4c, d).

Moreover, data from TCGA database confirmed positive correlation between IL-1β and HIF-2α mRNA expression in RCC (*p* < 0.001) (Fig. 4e and Table S2). Similarly, WB confirmed that HIF-2α expression was increased with the stimulation of IL-1β (Fig. 4f). In order to further detect whether the level of HIF-2α was upregulated through the secretion of IL-1β, we treated both RCC cells with IL-1β at different time and employed WB to analyze the expression of HIF-2α. The result elucidated that HIF-2α expression was induced along with IL-1β stimulating in a time-dependent manner (Supplementary Fig. 1B). Likewise, WB analysis of HIF-2α in both cells treated with different doses of IL-1β for 72 h also obtained the similar result (Supplementary Fig. 1C). Next, we investigated whether anti-IL-1β in RCC cells could interrupt the migration and invasion enhanced by B cells. As expected, IL-1β neutralizing antibody could inhibit the migration and invasion of 786-O and OSRC-2 cells. Meanwhile, B cells elevated migration and invasion were impaired as well (Fig. 4g, h). In addition, similar trends were obtained in the protein expression level of HIF-2α through WB (Fig. 4i).

In summary, IL-1β up-regulation secreting from B cells infiltration was the underlying mechanism by which B cells promoted metastasis and IL-1β was responsible for the upregulation of HIF-2α.

### AKT/P65 pathway was activated by IL-1β to promoted HIF-2α expression

**Fig. 5:**
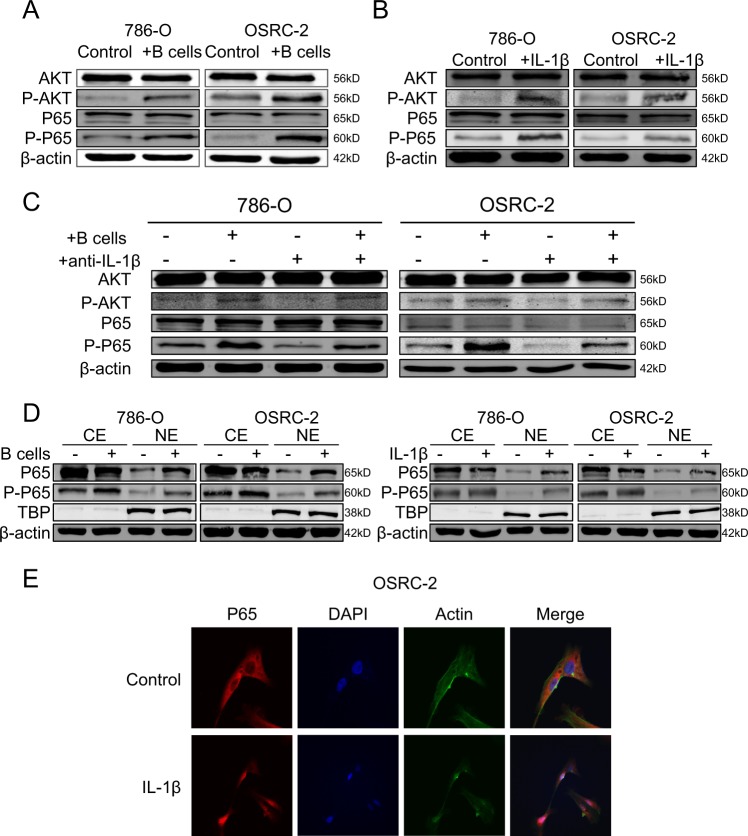
AKT/P65 pathway was activated by IL-1β to promoted HIF-2α expression. **a** After co-cultured with B cells, the expression of AKT, P-AKT, P65 and P-P65 in 786-O and OSRC-2 cells was detected by WB. **b** AKT, P-AKT, P65 and P-P65 expression with the stimuli of IL-1β was detected by WB. **c**. 786-O and OSRC-2 cells were co-cultured with B cells, pretreated with IL-1β neutralizing antibody or the combination. AKT, P-AKT, P65, and P-P65 expression was detected by WB. **d** Cytosol extracts (CE) and nucleus extracts (NE) were prepared and subsequently WB analysis was performed for P65 and P-P65 in 786-O and OSRC-2 after co-cultured with B cells or the stimulated with IL-1β. **e** Translocation of P65 was detected after stimulated with IL-1β in OSRC-2 by confocal assay.

Early studies indicated that IL-1β induced HIF-2α expression via AKT/P65 pathway^30^. Here, both RCC cells were co-cultured with B cells and then treated with several Pathway inhibitors respectively. Results demonstrated that the expression of HIF-2α was blocked after treated with LY294002 and Bay117082, which were inhibitors of AKT and P65 pathways, respectively (Supplementary Fig. 1D). Moreover, the protein content of AKT, the phosphorylation status of AKT, P65 and the phosphorylation status of P65 were measured by WB. As shown in (Fig. 5a), only the phosphorylation of AKT and the phosphorylation status of P65 were upregulated in 786-O and OSRC-2 cells after co-cultured with B cells. In parallel, the same tendency was observed after treated with IL-1β in both cells (Fig. 5b). In addition, we found that anti-IL-1β could abolish the induction of B cells on HIF-2α expression (Fig. 5c). As previously reported, P65 could translocate from cytoplasm to the nucleus when it was activated. To further explore the mechanism how P65 was activated by IL-1β to promoted HIF-2α expression, cytosol extracts (CE) and nucleus extracts (NE) were prepared, and WB assay was applied to substantiate that co-culture with B cells or treated with IL-1β both could promote the translocation of P65 and P-P65 into the nucleus (Fig. 5d). What’s more, result of confocal assays was also coincident with the above result (Fig. 5e).

### B cells induced HIF-2α upregulation activated DLL4 to initiate DLL4/Notch signals

**Fig. 6:**
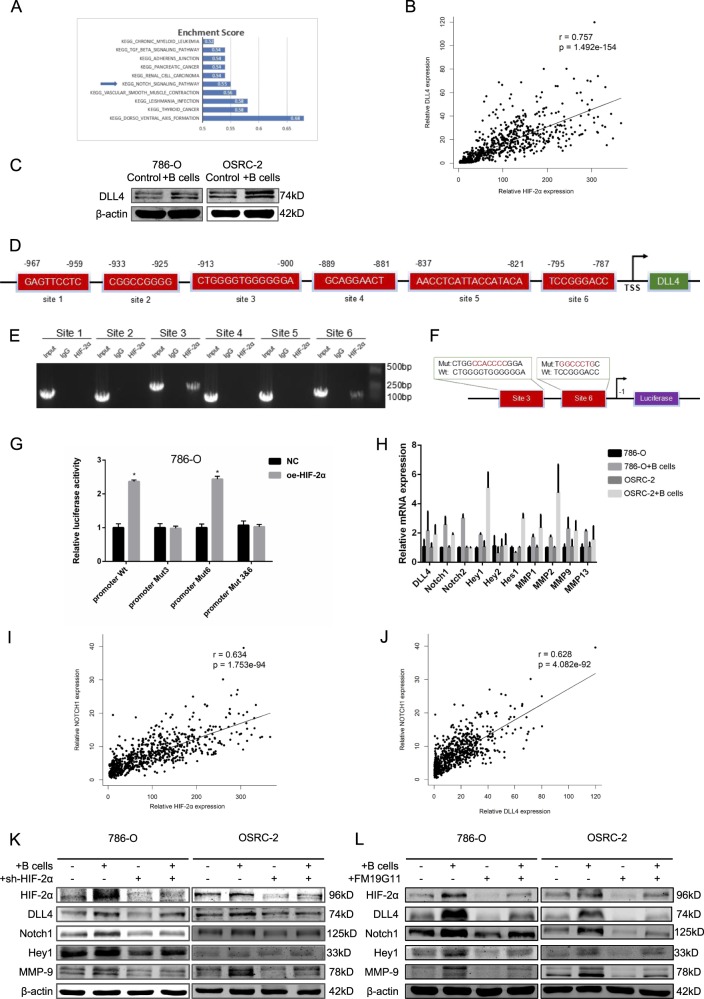
B cells induced HIF-2α upregulation activated DLL4 to initiate DLL4/Notch1 signals. **a** Top 10 KEGG pathways that were enriched for HIF-2α targets. **b** DLL4 mRNA expression and corresponding HIF-2α mRNA expression in RCC samples from TCGA dataset. **c** After co-cultured with B cells, the protein levels of DLL4 in 786-O and OSRC-2 cells was detected by WB. **d** Bioinformatic analysis of potential HIF-2α binding sites in DLL4 transcription start site (TSS). **e** Lysates of 786-O cells were subjected to ChIP assay and amplified by PCR reaction. **f** The schematic illustration of HIF-2α-wt and -mut in the DLL4 promoter. **g** Luciferase reporter assays in 786-O cells. **h** Genes in Notch signal were detected by qRT-PCR. **i**, **j** Notch1 mRNA expression and corresponding HIF-2α and DLL4 mRNA expression in RCC samples from TCGA dataset. **k**, **l** 786-O and OSRC-2 cells were co-cultured with B cells, pretreated with sh-HIF-2α (**k**) or HIF-2α (**l**) inhibitor FM19G11. HIF-2α, DLL4, Notch1, Hey1, and MMP-9 were detected by WB. *P < 0.05.

To investigate the potential mechanisms by which HIF-2α induced by B cells promoted the RCC progression, we first performed gene set enrichment analysis(GSEA) with Kyoto Encyclopedia of Genes and Genomes (KEGG) pathway database, and results revealed that Notch signal was most significantly enriched for the predicted targets of HIF-2α (Fig. 6a and Supplementary Fig. 1E). Moreover, previous study reported that DLL4 could promote RCC cell migration and invasion by activating intercellular Notch signaling^31^. Data in the UALCAN database revealed that mRNA expression of DLL4 were significantly higher in RCC tissues than in normal tissues in pan-cancer analysis (Supplementary Fig. 1F). Meanwhile, THE HUMAN PROTEIN ATLAS database showed that a majority of testicular cancers and a few cases of renal cancers showed moderate cytoplasmic positivity. Remaining cancers were negative or weakly stained (Supplementary Fig. 1G). Notably, we found data from TCGA database revealed that there was positive correlation between DLL4 and HIF-2α mRNA expression (Fig. 6b and Table S3). Whereby increased DLL4 expression at protein levels in 786-O and OSRC-2 cell lines co-cultured with B cells was confirmed by WB (Fig. 6c). Interestingly, results of qRT-PCR showed abolished DLL4 expression at mRNA levels in sh-HIF-2α group compared with control group in both RCC cell lines (Supplementary Fig. 1H). To further detect the molecular mechanism how HIF-2α increases DLL4 expression, we first applied public database to identify putative HIF-2α response elements (HREs) in the promoter region of DLL4 (Fig. 6d). We then applied the chromatin immunoprecipitation (ChIP) assay to verify its binding capacity. The results illustrated that HIF-2α could bind to two of the HREs (site3 and 6) located within 1 kb promoter region of DLL4 (Fig. 6e). Then we applied luciferase reporter assay to examine the HIF-2α transaction (Fig. 6f). The results of Luciferase assay revealed HIF-2α could increase the DLL4 promoter activity at site 3 in 786-O cells (Fig. 6g).

Intriguingly, qRT-PCR were employed to examine the expression of several genes in Notch signals and found that the expression of Notch1, Hey1, and MMP9 were significantly increased in 786-O and OSRC-2 cells after co-culturing with B cells (Fig. 6h). Next, data from TCGA database also attested that Notch1 mRNA expression was positive correlated with HIF-2α and DLL4 mRNA expression in both RCC cells (Fig. 6i, j, Tables S4 and S5).As expected, sh-HIF2α reversed the increased expression of HIF-2α, DLL4, Notch1, Hey1 and MMP9 in both RCC cells in the co-culture system (Fig. 6k). We applied FM19G11 as inhibitor of HIF-2α to repeat the above analysis and obtained similar results (Fig. 6l).

**Fig. 7:**
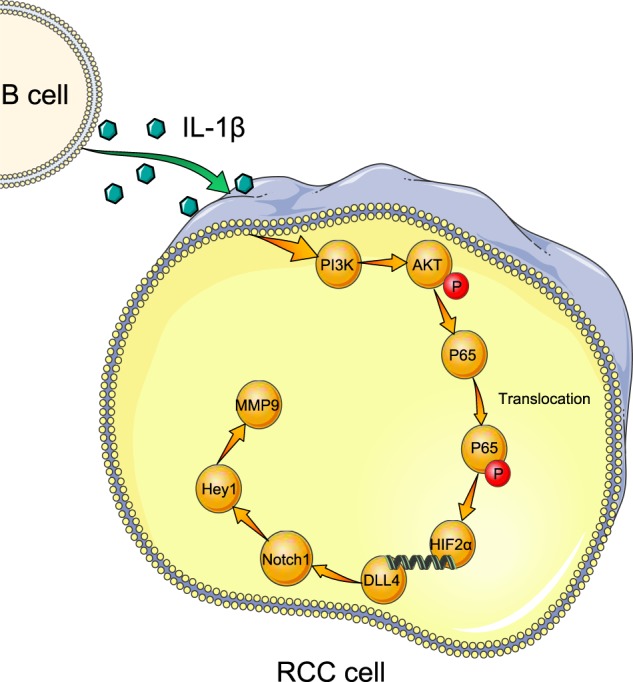
A Schematic illustration of the proposed model. Tumor-educated B cells promote renal cancer metastasis via inducing the IL-1β/HIF-2α/Notch1 signals.

Together, HIF-2α could increase the expression of DLL4 at the transcriptional level through directly binding to site 3 of DLL4 promoter region and then activate Notch1 signals. The HIF-2α/Notch1 signals might play key roles to mediate the infiltrating B cells-increased RCC cell migration and invasion (Fig. 7).

## Discussion

In this study, we first reported that more B cells were recruited in RCC tissues compared with the adjacent normal tissues, which facilitated RCC metastasis both in vitro and in vivo. Furthermore, B cells promoted RCC cell migration and invasion through the increase of HIF-2α, which was induced through the secretion of IL-1β. What’s more, AKT/P65 pathway was activated by IL-1β to promoted HIF-2α expression and B cells induced HIF-2α upregulation activated DLL4 to initiate DLL4/Notch1 signals. All these results supported the conclusion that TEB cells promote renal cancer metastasis via inducing the IL-1β/HIF-2α/Notch1 signals.

B cells played the imperative role in the TME from various tumor samples, particularly in the ccRCC, with a mean of 4% across samples^4^. However, their functional roles in the tumor progression remained controversial. B cells had been established as a metastasis-promoter in bladder cancer^9^ while opposite effects were also reported showing B cells could suppress other types of tumor development^32^. In the present study, we used in vitro migration assays, invasion assays, 3D invasion assays and in vivo tail vein injected mouse model to show that TEB cells could promote RCC metastasis.

B cells could secrete a number of cytokines after activation, including interleukin, chemokine, lymphotoxin and Interferon^33^. In addition, B cells required additional signaling beyond activation to become cytokine producers^34^. Thus, the additional signaling could be provided by the tumor microenvironment, which might play key roles in tumor progression due to its ability to enhance the migration and invasion of tumors. Accumulating evidences showed that IL-1β level was increased in a variety of human tumors^35^. Specifically, Sandra McAllister reported that IL-1β inflammatory response driven by primary breast cancer prevents metastasis-initiating cell colonization^32^. Regulatory B cells (Bregs), an important B cell subset producing IL-10, had been shown to contribute to autoimmune diseases, cancers, and chronic infections, which provided a possible mechanism how B cells turned into a tumor-promoter^36^. In our study, IL-1β produced by TEB promoted RCC metastasis through HIF-2α/Notch1 signal. Given its role of a metastasis-promoter in RCC, IL-1β might be considered as a urinary biomarker for the detection of mRCC.

It was widely believed that HIF was the central component in response to hypoxia^37^. Recently, increasing numbers of reports revealed that HIF was involved in the tumor growth and metastasis^38–40^, and we used to reported that hypoxia contributed to tumor progression in a HIF-2α-dependent manner in RCC^20^. Besides, an early study in osteoarthritis also suggested that IL-1β could hence NF-κB/HIF-2α activation through the PI3K/AKT pathway^30^. In this work, TEB facilitated IL-1β-induced RCC metastasis via activating AKT/P65/HIF-2α pathway.

Notch signaling was a conserved, widely expressed signal pathway that mediated various cellular processes in normal development including differentiation, proliferation, and apoptosis^41–43^. Intriguingly, in the progress of cancer, Notch1 might act as either an oncogene or a tumor suppressor gene depending on the type, stage and histotypic categories of tumor. Sun S, et al. found that the expression of Notch receptors was deregulated in the tumorigenesis of renal cell carcinoma^43^. Conversely, Qing Ai, et al. reported that high-level expression of Notch1 increased the risk of metastasis in T1 stage clear cell renal cell carcinoma^44^ and Jonas Sjölund revealed that inhibition of Notch signaling could suppress RCC growth^45^. The role of Notch1 in RCC had, to our knowledge, not been experimentally investigated previously. In our study, we testified that HIF-2α could increase the expression of DLL4 and then activate Notch1. The Notch1 signal might contribute to mediate the TEB-increased RCC cell migration and invasion in RCC TME.

In summary, our study showed that TEB was dramatically recruited in RCC, and served as an effective metastasis-promoter through IL-1β/HIF-2α/Notch1 signals in RCC patients. This finding might provide us a new strategy for the treatment of RCC in the future.

## Supplementary information

Supplementary Figure

Supplementary Figure Legends

Table S1

Table S2

Table S3

Table S4

Table S5

## Data Availability

Data and material is available at the website of Cell death and disease.
