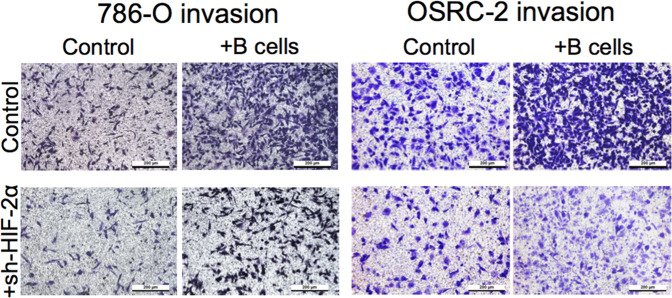# Correction: Tumor-educated B cells promote renal cancer metastasis via inducing the IL-1β/HIF-2α/Notch1 signals

**DOI:** 10.1038/s41419-022-04878-7

**Published:** 2022-04-29

**Authors:** Saiyang Li, Chi Huang, Guanghui Hu, Junjie Ma, Yonghui Chen, Jin Zhang, Yiran Huang, Junhua Zheng, Wei Xue, Yunfei Xu, Wei Zhai

**Affiliations:** 1 Department of Urology, Shanghai Tenth People’s Hospital, Nanjing Medical University, 211166 Nanjing, China; 2 Department of Urology, the Affiliated Changzhou Maternity and Child Health Care Hospital of Nanjing Medical University, 16 DingxiangRoad, Zhonglou District, 213000 Changzhou, Jiangsu China; 3 Department of Urology, Shanghai Tenth People’s Hospital, School of Medicine in Tongji University, 200072 Shanghai, China; 4 Department of Urology, Shanghai Pudong Hospital, Fudan University Pudong Medical Center, 201399 Shanghai, China; 5 Department of Urology, Renji Hospital, School of Medicine in Shanghai Jiao Tong University, 160 Pujian Road, Pudong District, 200127 Shanghai, China; 6 Department of Urology, Shanghai First People’s Hospital, School of Medicine in Shanghai Jiao Tong University, 200080 Shanghai, China

**Keywords:** Renal cell carcinoma, Cell invasion

Correction to: *Cell Death and Disease* 10.1038/s41419-020-2355-x, published online 02 March 2020

The original version of this article unfortunately contained an error in figure 3D. The authors apologize for these unintentional mistakes which occurred due to transfer a large amount of data between two institutes (the Affiliated Changzhou Maternity and Child Health Care Hospital of Nanjing Medical University and Shanghai Tenth People’s Hospital) with three different computers during figures preparation from three co-first authors. The correct figure can be found below.