# Supplementary figures and images for: Tumor-educated B cells promote renal cancer metastasis via inducing the IL-1β/HIF-2α/Notch1 signals
Source: Cell Death Dis. 2020 Mar 2;11(3):163. doi: 10.1038/s41419-020-2355-x (PMC7052134; doi:10.1038/s41419-020-2355-x)

# Figure 1

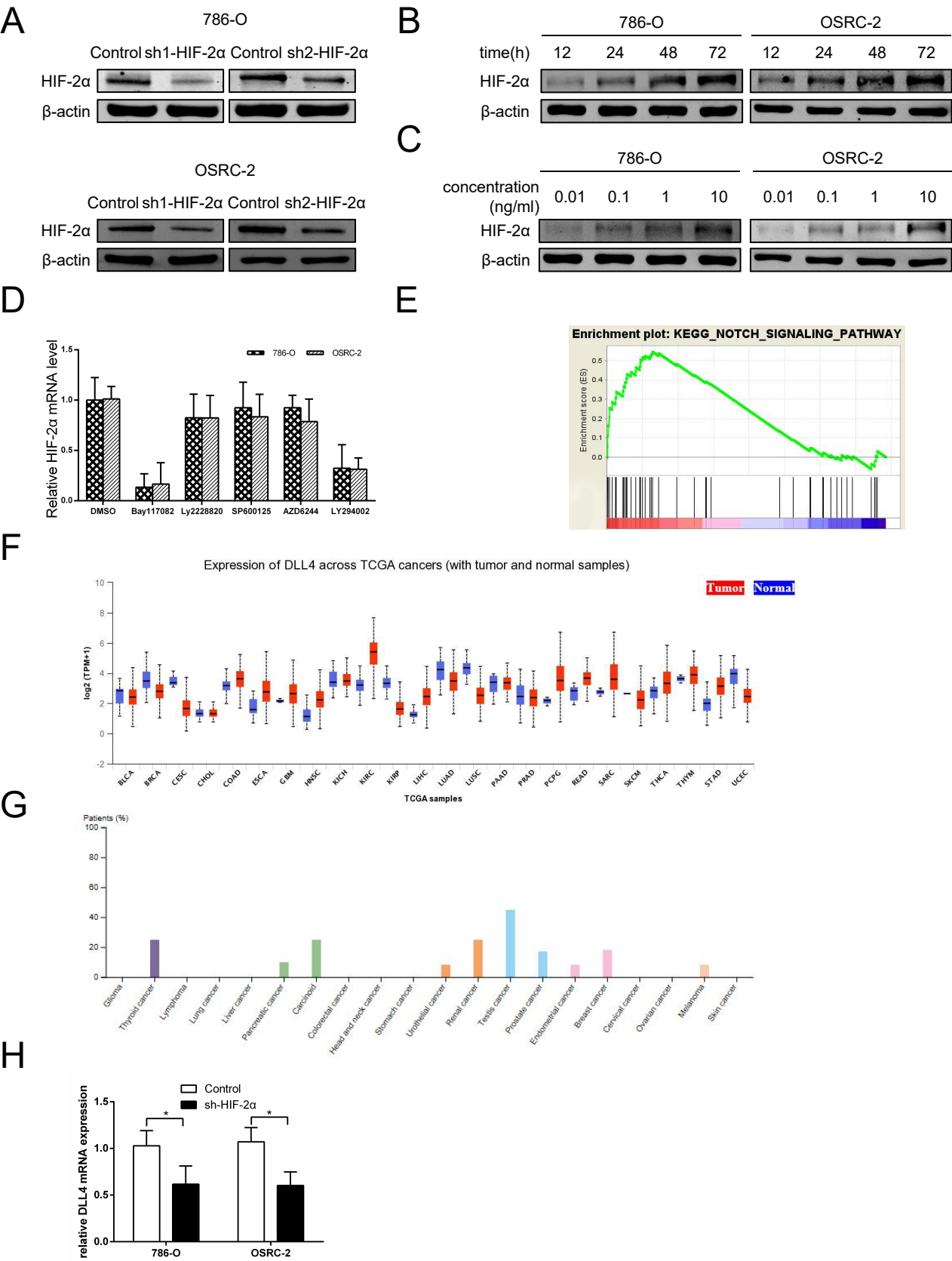

Supplement: Supplementary file 1 — Supplementary Figure [file 41419_2020_2355_MOESM1_ESM.pdf]
