# Supplementary material for: Tumor-educated B cells promote renal cancer metastasis via inducing the IL-1β/HIF-2α/Notch1 signals
Source: Cell Death Dis. 2020 Mar 2;11(3):163. doi: 10.1038/s41419-020-2355-x (PMC7052134; doi:10.1038/s41419-020-2355-x)
Supplement: Supplementary file 2 — Supplementary Figure Legends [file 41419_2020_2355_MOESM2_ESM.doc]

**Supplementary Figure1** A. The protein levels of HIF-2α in 786-O and OSRC-2 cells was detected by WB after treated with sh1-HIF-2α and sh2-HIF-2α. B. The protein levels of HIF-2α in 786-O and OSRC-2 cells was detected by WB after stimulated with IL-1β at 12, 24, 48 and 72 hours. C. The protein levels of HIF-2α in 786-O and OSRC-2 cells was detected by WB after stimulated with IL-1β at 0.01, 0.1, 1, 10ng/ml. D. The mRNA levels of HIF-2α in 786-O and OSRC-2 cells was detected by qRT-PCR after treated with pathway inhibitors. E. Notch signal was significantly enriched for the predicted targets of HIF-2α from gene set enrichment analysis(GSEA) with Kyoto Encyclopedia of Genes and Genomes (KEGG) pathway database. F. Boxplot showing relative mRNA expression of DLL4 in normal and tumor samples. G. Boxplot showing relative protein expression of DLL4 in normal and tumor samples. H. The mRNA levels of DLL4 in 786-O and OSRC-2 cells was detected by qRT-PCR after treated with sh-HIF-2α.
